# Comprehensive analyses of brain cell communications based on multiple scRNA‐seq and snRNA‐seq datasets for revealing novel mechanism in neurodegenerative diseases

**DOI:** 10.1111/cns.14280

**Published:** 2023-06-02

**Authors:** Chunlong Zhang, Guiyuan Tan, Yuxi Zhang, Xiaoling Zhong, Ziyan Zhao, Yunyi Peng, Qian Cheng, Ke Xue, Yanjun Xu, Xia Li, Feng Li, Yunpeng Zhang

**Affiliations:** ^1^ College of Bioinformatics Science and Technology Harbin Medical University Harbin China

**Keywords:** Alzheimer's disease, bioinformatic platform, brain, cell communications

## Abstract

**Aims:**

Complex cellular communications between glial cells and neurons are critical for brain normal function and disorders, and single‐cell level RNA‐sequencing datasets display more advantages for analyzing cell communications. Therefore, it is necessary to systematically explore brain cell communications when considering factors such as sex and brain region.

**Methods:**

We extracted a total of 1,039,459 cells derived from 28 brain single‐cell RNA‐sequencing (scRNA‐seq) or single‐nucleus RNA‐sequencing (snRNA‐seq) datasets from the GEO database, including 12 human and 16 mouse datasets. These datasets were further divided into 71 new sub‐datasets when considering disease, sex, and region conditions. In the meanwhile, we integrated four methods to evaluate ligand–receptor interaction score among six major brain cell types (microglia, neuron, astrocyte, oligodendrocyte, OPC, and endothelial cell).

**Results:**

For Alzheimer's disease (AD), disease‐specific ligand–receptor pairs when compared with normal sub‐datasets, such as SEMA4A‐NRP1, were identified. Furthermore, we explored the sex‐ and region‐specific cell communications and identified that WNT5A‐ROR1 among microglia cells displayed close communications in male, and SPP1‐ITGAV displayed close communications in the meninges region from microglia to neurons. Furthermore, based on the AD‐specific cell communications, we constructed a model for AD early prediction and confirmed the predictive performance using multiple independent datasets. Finally, we developed an online platform for researchers to explore brain condition‐specific cell communications.

**Conclusion:**

This research provided a comprehensive study to explore brain cell communications, which could reveal novel biological mechanisms involved in normal brain function and neurodegenerative diseases such as AD.

## Graphical abstract

We performed a multidimensional analysis of single‐cell interactions by analyzing a total of 1,039,459 cells derived from 28 brain scRNA‐seq transcriptome datasets, including 12 human and 16 mouse datasets. And we also constructed a comprehensive online database for exploring condition‐specific cell interactions, which aids in clinical studies of brain disorders, such as neurodegenerative diseases.

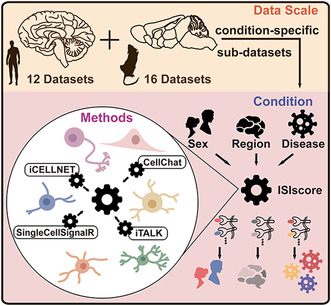

## 1 INTRODUCTION

Neurodegenerative diseases are a broad group of central nervous system disorders with pathological features including brain atrophy, plaques, neurofibrillary tangles, and aggregates.^1^, ^2^, ^3^ It has been reported that atrophy in the human brain is mainly in the temporal and frontal lobes, with strong regional heterogeneity.^4^ In the meanwhile, there exists sex tendentiousness among different types of neurodegenerative diseases. For example, Alzheimer's disease (AD) is more common in women than in men^5^; and women are 2–3 times more likely to have major depressive disorder (MDD) than men^6^; while Parkinson's disease (PD) and amyotrophic lateral sclerosis (ALS) predominantly affect men^7^, ^8^; also spinal and bulbar muscular atrophy (SBMA) affects only males.^9^ Therefore, it is necessary to consider sex dimorphism and regional heterogeneity in brain when exploring the biological mechanism involved in neurodegenerative diseases.

Multicellular life depends on the coordination of cellular activity, which in turn depends on cell–cell communications between different types of cells.^10^, ^11^, ^12^ Cellular communications coordinate the development, homeostasis, and single‐cell function of organisms.^13^ It is now widely accepted that cell–cell interactions exist across the majority of cell types in the brain immune microenvironment. Single‐cell RNA sequencing (scRNA‐seq), which examined the transcriptomic profile of single cell with next‐generation sequencing technologies, provided a better understanding of the function of an individual cell in the context of its microenvironment.^14^ For frozen brain samples, single‐nucleus RNA sequencing (snRNA‐seq) is also an important strategy, which can address these samples that cannot be readily dissociated into a single‐cell suspension and minimizes the issues caused by the dissociation procedure. ScRNA‐seq and snRNA‐seq data provided us with a more precise resource for understanding the cellular heterogeneity of the brain and how cells interact within their microenvironment.^14^, ^15^, ^16^ Utilizing the scRNA‐seq data, Jiang et al. performed the cell–cell interaction analyses in cancers to explore the mechanisms underlying response or resistance to anti‐PD‐1 therapy and investigated the relative difference of interaction changes.^17^ In the meanwhile, a cluster of T cells that exhibited an expression pattern of ligand/receptor were identified, and these cells displayed increased expression of survival‐related genes. These findings based on cell–cell interaction offered abundant clues for potential strategies to improve immunotherapy. The brain single‐cell atlas paints a unique cellular‐level view of transcriptome alterations associated with normal and disordered brains and significantly improves our understanding of the pathogenesis of neurodegenerative diseases, such as AD.^18^

For dissecting the function/dysfunction of higher heterogeneous cells in the AD brain at the single‐cell level, Jiang et al. obtained 17 existing human and mouse AD scRNA‐seq and snRNA‐seq datasets from GEO and Synapse databases.^19^ Considering species, gender, brain region, age stage, and disease conditions, sub‐datasets were formed. Based on each sub‐dataset, the differentially expressed genes and cell‐type‐specific regulons were identified for in‐depth analysis of heterogeneous regulatory mechanisms. Finally, these researchers developed an integrated database named scREAD for exploring brain tissues with AD and mouse models with AD pathology. However, scREAD only contained AD disease, and without consideration of other neurodegenerative diseases such as MS. Moreover, cell communications when considering AD gender and region conditions were not explored, and the dysregulation of ligand–receptor interactions between immune cells and neuron was biologically critical in brain immune function.

In recent years, many laboratories have developed kinds of algorithms based on scRNA‐seq or snRNA‐seq datasets for cell communication studies, such as CellChat, a method for generalized ligand–receptor models based on hash equations^20^; iCELLNET, a method for calculating the overall cell communication score by summing the product of all ligand–receptor pairs between two groups^21^: iTALK, which identifies cell communication scores between different clusters by enumerating differentially expressed ligands and receptors^22^; and SingleCellSignalR, which uses regularized ligand–receptor products to evaluate the degree of cell communications,^23^ etc. There exist different differences between results from different algorithms, and it is necessary to perform the integration based on multiple methods.

In the current study, we explored the mechanisms of normal brain function and neurodegenerative diseases based on scRNA‐seq data from normal and disordered brains. Our study mainly explored the biological mechanisms underlying brain immune from the perspective of ligand–receptor interactions among cells. We believe that the neurodegenerative diseases is affected by many factors, and among these, the effect of cell–cell communications has not been systemically explored thus far. Therefore, we integrated multiple scRNA‐seq and snRNA‐seq datasets to examine the differences in brain cell communications, which was calculated by four common methods when considering different conditions such as disease, sex, and brain regions. Comprehensive analyses of brain cell communications identified novel ligand–receptor pairs involved in disease formation, sex difference in normal brain as well as regional heterogeneity. Take AD as an example, we explored the AD subtypes based on cell communications, which displayed abnormally in AD compared to normal conditions. In the meanwhile, the model based on ligand–receptor pairs displayed predictive performance using multiple bulk transcriptome datasets. Finally, we developed a comprehensive bioinformatic platform (http://bio‐bigdata.hrbmu.edu.cn/BrainCelnt), which aided in exploring ligand–receptor interactions in normal and disordered brains.

## 2 MATERIALS AND METHODS

### 2.1 Brain dataset resource

By manually searching for keywords: ‘brain’, ‘scRNA‐seq’, ‘snRNA‐seq’, ‘single‐cell’, ‘RNA sequencing’ from Gene Expression Omnibus (GEO) database, we collected a total of 28 brain scRNA‐seq and snRNA‐seq datasets for both human and mouse (human: 12, mouse: 16, see Tables S1 and S2). For human datasets, 11 datasets were derived from normal samples, three datasets were derived from AD samples and two datasets were derived from MS samples; For mouse datasets, 16 datasets were derived from normal samples and 2 datasets were derived from AD samples. And, the sample clinical information such as brain regions and sex was also obtained from the original studies. For brain regions, a total of 12 regions were collected, including hippocampus, frontal lobe, parietal lobe, meninges and other extracerebral areas, temporal lobe, occipital lobe, diencephalon, cerebellum, cortex, cortex+diencephalon, cortex+brainstem, and brainstem.

To test the performance of predictive model based on AD‐specific ligand–receptor pairs, we furthermore obtained four bulk transcriptome datasets of AD samples from GEO database. GSE1297^24^ contains hippocampal gene expression derived from 9 controls and 22 samples with different severity of AD samples; GSE5281^25^ contains gene expression in multiple regions from 74 controls and 87 AD samples; GSE29378^26^ contains hippocampal gene expression from 32 controls and 31 AD samples; GSE118553^27^ contains 100 controls, 134 asymmetric AD subjects and 167 AD samples; GSE159699^28^ contained 18 controls and 12 AD samples.

### 2.2 scRNA‐seq and snRNA‐seq dataset processing

All scRNA‐seq and snRNA‐seq datasets were processed using *Seurat* in R (v.4.0.3) and each dataset was analyzed separately. In detail, cells with mitochondrial gene over‐expression were filtered out (percentage. mt >20%). NormalizeData() was used to log‐normalize the data and scale it to 10,000 transcripts per cell using Scale(). The FindVariableFeatures() function was used to identify the first 2000 variable genes. RunPCA() implemented Principal Component Analysis (PCA) for dimensionality reduction. ElbowPlot() determined the principal components, and FindClusters() function implemented the shared nearest neighbor (SNN) to identify clusters. Using the *clustree* package^29^ for each dataset to determine the optimal resolution, and it can visualize the relationship between clusters at multiple resolutions.

### 2.3 Cell‐type annotation

Six major intracerebral cell types were annotated, namely astrocytes, endothelial cells, microglia, neurons, oligodendrocyte progenitor cells (OPC), and oligodendrocytes, with the remaining cells being annotated as ‘other cells’. And, we used marker genes specific to each cell type as described in the CellMarker database.^30^ Take the human as an example, these markers included but were not limited to, astrocytes: GFAP; endothelial cells: CLDN5; microglia: CX3CR1 and CSF1R; neuronal cells: SLC17A7; oligodendrocyte progenitor cells: PDGFRA; oligodendrocytes: MAG and OLIG2. The detailed marker genes for all cell types are provided in Table S3. Specifically, the marker genes for each cell cluster were found using the FindAllMarker() function in the *Seurat* package and compared against the cell type‐specific marker genes.

### 2.4 Calculating integrated ligand–receptor interaction score

For each scRNA‐seq and snRNA‐seq dataset, the original dataset could be further divided into different sub‐datasets when considering different clinical factors such as sex, brain region, and disease condition. Take the GSE118257 as an example, this dataset could be divided into two sub‐datasets: GSE118257‐MS and GSE118257‐normal. When considering sample sex information, the sub‐dataset GSE118257‐MS could further be divided into two sub‐datasets: GSE118257‐MS‐Male and GSE118257‐MS‐Female. The detailed sub‐dataset information is shown in Table S4. For each sub‐dataset, we utilized four different methods, CellChat,^20^ iTALK,^22^ iCELLNET,^21^ and SingleCellSignalR,^23^ to calculated the ligand–receptor score with the default parameter in each method. As iCELLNET and iTALK did not provide a database of mouse ligands and receptors, we used the *homologene* package to homologate mouse genes to human genes. To obtain the consistent score, we defined the ligand–receptor pairs that existed in four methods higher score. In this study, the original ligand–receptor score was normalized to [0, 1] using scale() function for each method, and zero was assigned to the pair which was not existed in corresponding method. To improve the cell communications, the mean value of four methods was defined as ISIscore (Integration Standardized Interaction score) for each ligand–receptor pair. Higher ISIscore displayed stronger communications between ligand–receptor pairs in corresponding brain sub‐dataset.

### 2.5 Evaluating cell communication differences

We evaluated the differences of ligand–receptor pairs between different sub‐datasets when considering disease, sex, or brain region conditions. Take the AD (disease) condition as an example, the ISIscore was compared between AD and normal sub‐datasets for each ligand–receptor pair. The ligand–receptor pairs with ISIscore equal to zero for all disease and normal sub‐datasets were removed for the following analysis. And the difference in ISIscore was calculated using Wilcoxon rank‐sum test, and the results with *p*‐value <0.05 were considered significant. Similarly, when considering different sub‐datasets comparisons, we identified the disease‐specific (AD or MS), sex‐specific (AD‐male, AD‐female, normal‐male, or normal‐female), and region‐specific cell communications. For two sub‐datasets (GSE147528‐AD and GSE126836‐normal), the significant ligand–receptor pairs with log ISIscore ratio between AD and normal more than three were considered as AD‐specific cell communications.

### 2.6 AD sub‐groups classification framework

Gene interactions including ligand–receptor pairs are rarely disrupted in normal condition and are extensively disrupted in lesions.^31^, ^32^ And there might exist cell communication heterogeneity between AD samples. Therefore, we utilized the edge perturbation‐based approach developed by Chen^33^ to explore and identify novel subtypes using AD specific ligand–receptor pairs, which included three main steps: (i) Based on the AD bulk transcriptome dataset, we firstly conversed the ligand–receptor expression matrix into a gene expression rank matrix: expression value of each gene was converted to its rank for each sample. (ii) For each AD‐specific ligand–receptor pair, the rank of the ligand was subtracted from the rank of the receptor to obtain final value of ligand–receptor pair. And then, a ligand–receptor edge matrix was constructed. (iii) For normal expression matrix, we ranked the genes by the mean gene expression value across all normal samples and formed edge rank matrix according to the step ii. (iv) Finally, we formed a ligand–receptor perturbation matrix by subtracting normal matrix from AD matrix. Based on the perturbation matrix, consensus clustering was performed using *ConcensusClusterPlus* package to infer AD subtypes.^34^ The clustering method was performed to select PAM with sample and gene ratio of 0.8 and 0.8, respectively, and this process was performed 1000 times at random to obtain final clusters.

### 2.7 Bioinformatic platform construction

For displaying condition‐specific brain cell communications for users, we developed a bioinformatic platform named BrainCelnt. BrainCelnt was developed using Struts2, Java Server Pages (JSP), and runs under RedHat 6.4. The database has relatively strong compatibility and has been tested on major web browsers (e.g. Microsoft Edge, Firefox, Chrome, Safari). MySQLv5.6.25 is used for data storage and runs on Apache web server v6.0.44. Dynamic HTML pages are implemented using JSP and JavaScript and dataset tables are implemented using the JQuery plugin.

## 3 RESULTS

### 3.1 The workflow of this study

**FIGURE 1:**
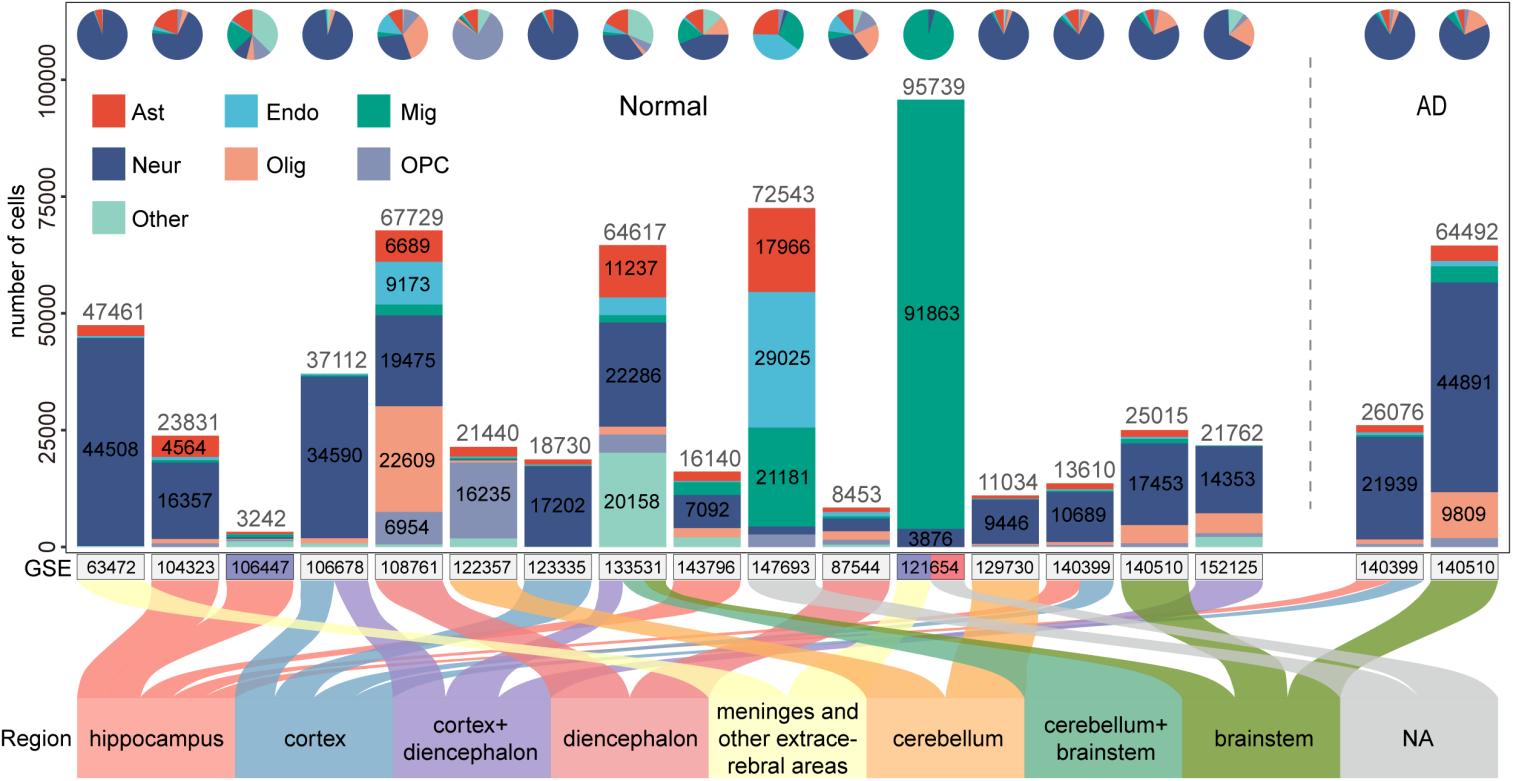
Human scRNA‐seq and snRNA‐seq dataset resource. The bar and pie charts in the top indicated the number of cells in each dataset and the proportion of cell types. The vertical coordinate indicated the number of cells, and the horizontal coordinate indicated datasets, where the color of the horizontal coordinate represented whether there existed gender information (male: blue, female: red). The lower part of the Sankey plot displayed the brain region information for all datasets.

The overall workflow of this study is shown in Figure S1. First, a total of 28 sets of mouse and human scRNA‐seq (and snRNA‐seq) datasets were obtained from the GEO database. Each dataset was processed through a standard single‐cell processing pipeline and cell annotation. After quality control, 430,181 cells were obtained for human and 609,278 cells were obtained for mouse. The detailed number of cell types and sample clinical information for each human and mouse dataset are shown in Figure 1, Figure S2, and Table S4. Considering different conditions, these 28 datasets were further divided into 71 sub‐datasets (see Table S5). For each sub‐dataset, we integrated four methods to explore the cell communications and calculated the ISIscore (see Materials and Methods). Based on the ISIscore, the condition‐specific (including disease‐, sex‐, and region‐specific) ligand–receptor pairs were identified. For exploring novel biological mechanism involved in AD, we constructed a predictive model based on AD‐specific cell communications and defined the sub‐groups for AD samples.

### 3.2 AD‐specific brain cell communications

**FIGURE 2:**
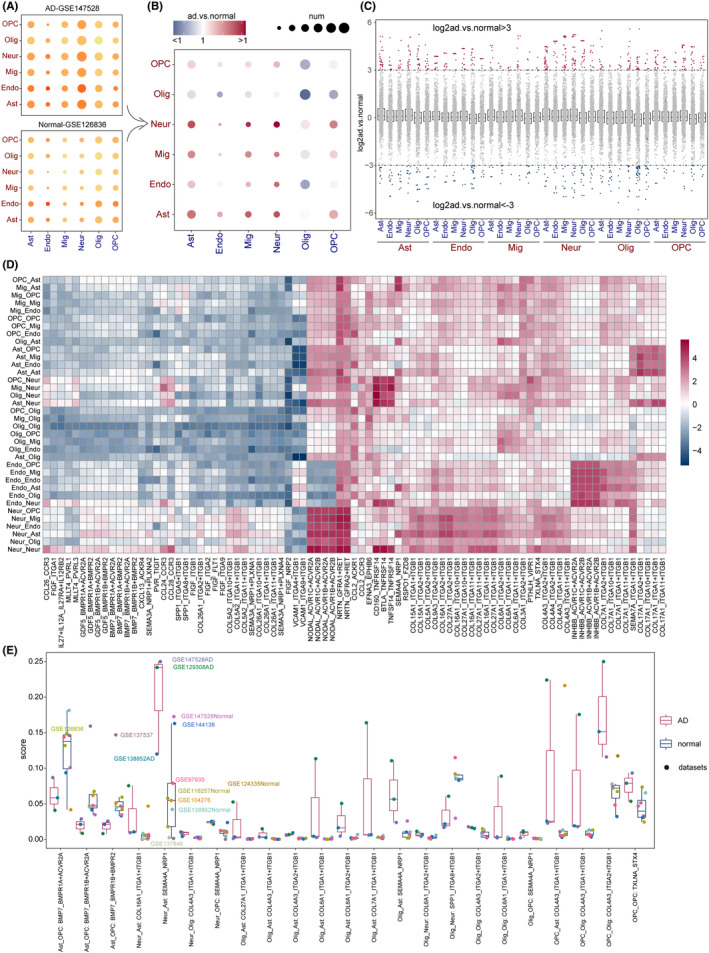
Human AD‐specific cell communications. (A) The total number of ligand–receptor pairs and the sum of ISIscore in the AD sub‐dataset (GSE147528‐AD) and normal sub‐dataset (GSE126836‐normal). The size of the dot represented the number of ligand–receptor pairs, and the color of the dot represented the sum of ISIscore. (B) Significant ligand–receptor pairs with Wilcoxon rank‐sum test and the ratio of ISIscore between AD and normal sub‐dataset. The size of the dots indicated the number of significant ligand–receptor pairs for cell communications. (C) AD‐ and normal‐specific ligand–receptor pairs with |log2(AD/normal)| > 3 and <−3. The horizontal coordinates indicated cell communications and the dots indicated ligand–receptor pairs. (D) The ratio of ISIscore of ligand–receptor pairs for cell communications between these two sub‐datasets. (E) Significant AD‐ and normal‐specific ligand–receptor pairs when considering all AD and normal sub‐datasets.

Firstly, we chose two sub‐datasets (GSE147528‐AD and GSE126836‐normal) as an example to identify AD‐specific ligand–receptor pairs. The number of ISIcore sums for all cell communications and the number of pairs in which communications occurred is shown in Figure 2A. It was observed that these cell pairs differed to some extent in both the intensity and number of communications. Notably, the number of ligand–receptor pairs was significantly higher in the AD sub‐dataset than in the normal sub‐dataset. Furthermore, we screened significant ligand–receptor pairs and displayed the sum of the ISIscore results for each cell communications in Figure 2B. More ligand–receptor communications among neuron, microglia, astrocytes, and endothelial cells in the AD than normal suggested that these cells communicated with each other more closely in the AD condition. The log ISIscore ratio between AD and normal for all ligand–receptor pairs is shown in Table S6. By setting strict cutoff (3 or 1/3), we respectively identified the AD‐ and normal‐specific ligand–receptor pairs for all cell communications (see Figure 2C). For example, the ligand INHBB and receptor ACVR1C+ACVR2A complex displayed more closely communications in AD when endothelial cells acted as senders, whereas the ligand FIGF and receptor NRP2 displayed close communications in normal when neurons acted as receivers (see Figure 2D).

Based on all AD and normal sub‐datasets, we further identified the significant ligand–receptor pairs (Figure 2E). It was observed that ligand BMP7 and receptor BMPR1A+ACVR2A, BMPR1B+ACVR2A, and BMPR1B+BMPR2 displayed close communications in normal from astrocytes to OPC. Previous studies have revealed that BMP family was involved in neurogenesis, axon pathfinding, and dendritic branching.^35^, ^36^ Additional evidence demonstrated that AD pathology involved reduced expression of BMP7.^37^ The ligand–receptor SEMA4A_NRP1 displayed higher score in AD from multiple cell communications, including neuron‐astrocyte, neuron‐OPC, oligodendrocyte‐astrocyte, and oligodendrocyte‐OPC. And it has been confirmed that NRP1 is involved in the inflammatory process in AD.^38^ Similarly, we also identified AD‐ and normal‐specific ligand–receptor pairs from the mouse datasets (Figure S3).

### 3.3 AD prediction and subgroup analysis based on cell communications

**FIGURE 3:**
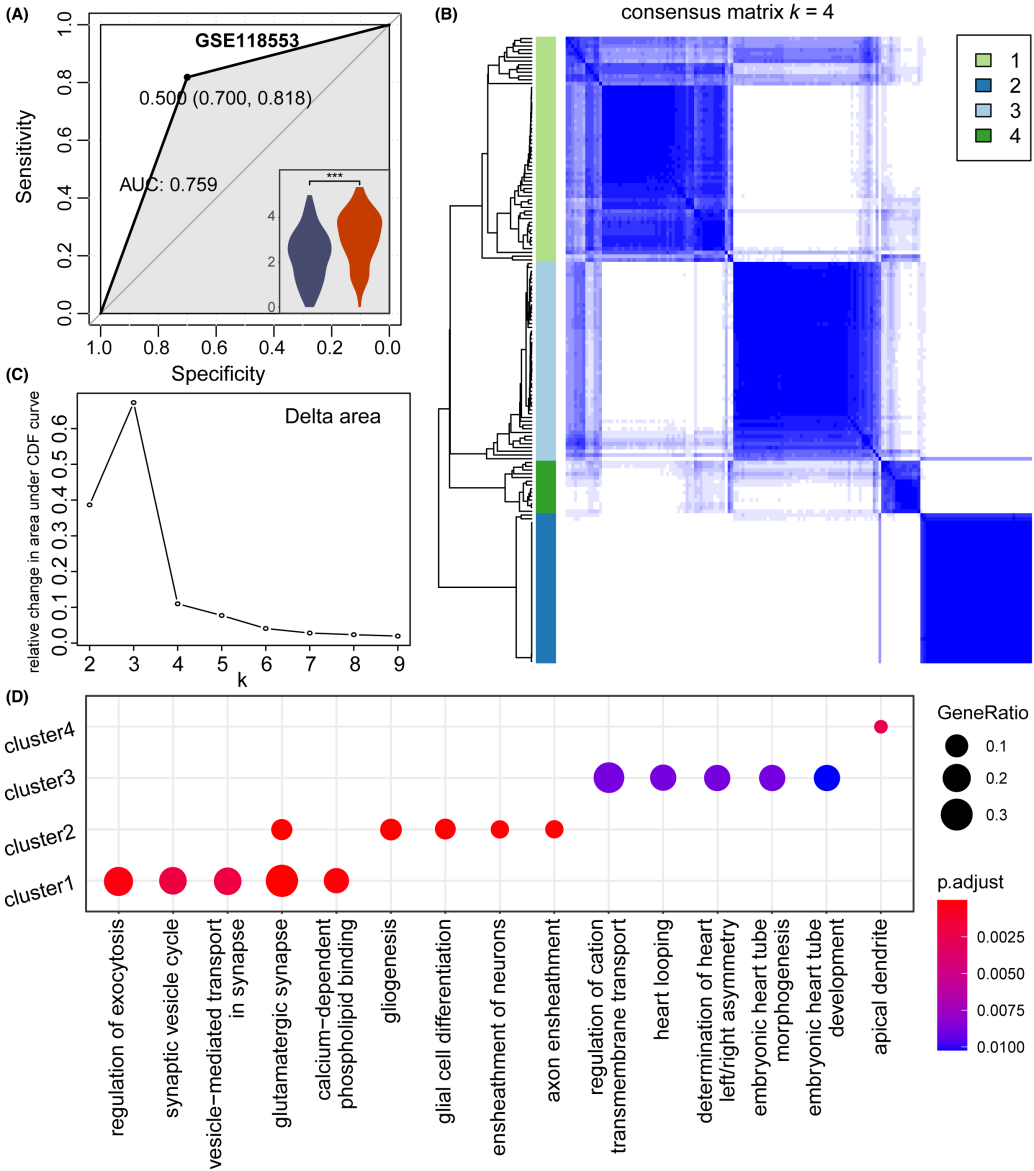
AD subtype analysis based cell communications (GSE118553). (A) ROC curves of model using AD ligand–receptor pairs in Figure 2C. The lower right‐hand corner showed the distribution of perturbation values for log2‐transformed reciprocal pairs in the normal and AD samples. (B) Heatmap of the optimal consensus matrix based on AD ligand–receptor pairs. Rows and columns indicated AD samples. (C) The Delta area plot showed the relative change in area under the CDF curve. Area change slowed down when *K* = 4. (D) The top 5 Gene Ontology (GO) terms for the four subtypes identified above.

As shown in Figure 2C, a total of 129 genes were obtained as AD specific (see Table S7). To test the predictive performance of AD‐specific markers, XGBoost model was used to test whether these genes could predict AD occurrence based on one bulk transcriptome dataset (GSE118553). As shown in Figure 3A, these AD‐specific ligand–receptor displayed performance on AD prediction with area under the curve (AUC) values equal to 0.759, with random 75% samples as training set and remaining 25% samples as testing set. In the meanwhile, the AD samples exhibited higher expression value of these genes than normal samples. Furthermore, to test the predictive robustness, we obtained more AD bulk transcriptome datasets (see Materials and Methods) and confirmed the performance of this predictive model (see Figure S4).

Based on the AD‐ and normal‐specific ligand–receptor pairs, we further performed subgroup analysis for exploring the AD heterogeneity (see Materials and Methods, Figure 2C and Table S7). The heatmap showed four clear clusters, and the delta area map also clearly no longer decreased at *K* = 4, suggesting that AD samples can be divided into four subgroups based on cell communications (Figure 3B,C). After excluding asymmetric patients, the 167 AD samples were divided into four subgroups, including 60 subgroup1, 40 subgroup2, 53 subgroup3, and 14 subgroup4. By representative genes, the Gene Ontology (GO) enrichment analysis was performed. As shown in Figure 3D, most of the GO terms enriched in subgroup1 were related to synapses, such as synaptic vesicle cycle, vesicle‐mediated transport in synapse, glutamatergic synapse. In addition, the regulation of exocytosis was also enriched. Some GO terms associated with glial cells were enriched in subgroup2, such as glial cell differentiation, gliogenesis, which may suggest that the pathological features of subgroup2 are associated with early glial cell formation, possibly in the formative stages of AD (see Table S8). In the meanwhile, the subgroup‐specific ligand–receptor pairs were also identified, such as EFNA3_EPHA6, SST_SSTR2, and RSPO3_FZD8 in subgroup2 (see Figure S5).

### 3.4 Sex‐specific cell communications in normal brain

**FIGURE 4:**
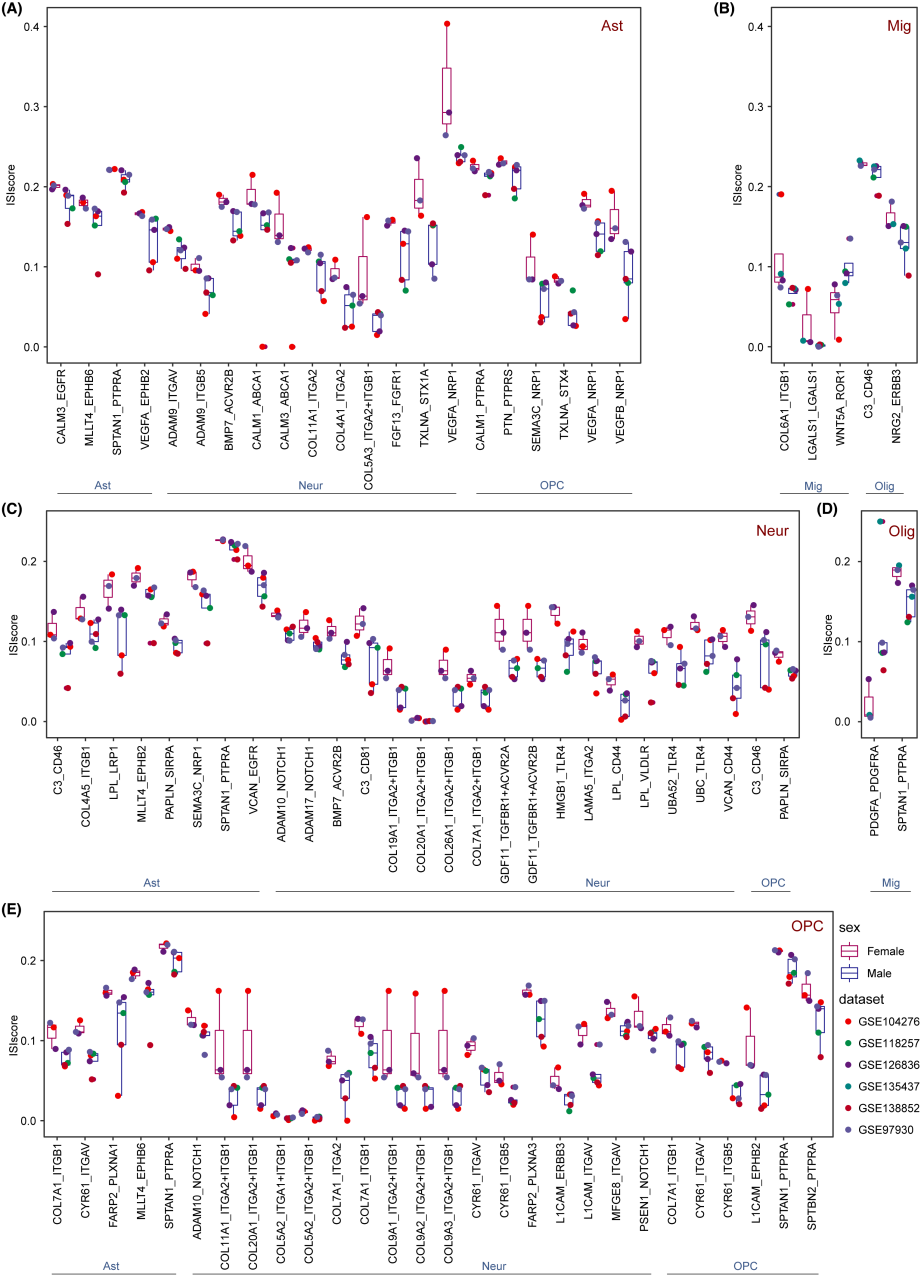
Human sex‐specific cell communications. Sex‐specific ligand–receptor pairs for (A) astrocytes, (B) microglia, (C) neuron, (D) oligodendrocytes, and (E) oligodendrocyte progenitor cells as the sending cell. The colors of the boxes represented the different sex (male and female) and the colors of the dots represented the datasets. Red text indicated sending cells, and blue text indicated receiving cells.

There exists sexual dimorphism in the biological mechanism of the brain, and there are also some sex differences in the interactions between brain cells.^39^ Therefore, we further explored the sex‐specific ligand–receptor pairs using normal sub‐datasets, which was similar to disease analysis. The ligand–receptor MLLT4_EPHB6 displayed higher score in female from multiple cell communications including astrocyte‐oligodendrocytes and astrocytes‐astrocytes (Figure 4A). Previous study has shown that females expressed higher levels of adrenergic receptor B6 (EphB6) in certain brain regions compared to males.^40^ In the meanwhile, NRP1 was also involved in significant sex‐specific ligand–receptor pairs. In the microglia communications, the ligand–receptor WNT5A_ROR1 displayed higher score in males (Figure 4B). And Wnt5a was confirmed to act as an important morphogenetic factor in sexual development.^41^ And for microglia‐oligodendrocytes communications, C3_CD46 and NRG2_ERBB3 displayed higher score in female (Figure 4B). Sex‐specific ligand–receptor pair C3_CD46 was also observed from neurons to astrocytes and OPC (Figure 4C). For oligodendrocytes‐microglia communications, PDGFA_PDGFRA and SPTAN1_PTPRA were considered significantly different in sex condition. The ligand–receptor PDGFA_PDGFRA displayed close communications in males and SPTAN1_PTPRA displayed communications in females (Figure 4D). From OPC to multiple cell types (astrocytes, neurons, and OPC), there existed sex‐specific ligand–receptor pairs CYR61_ITGAV and COL7A1_ITGB1, with stronger interaction scores in females (Figure 4E).

### 3.5 Brain region‐specific cell communications

**FIGURE 5:**
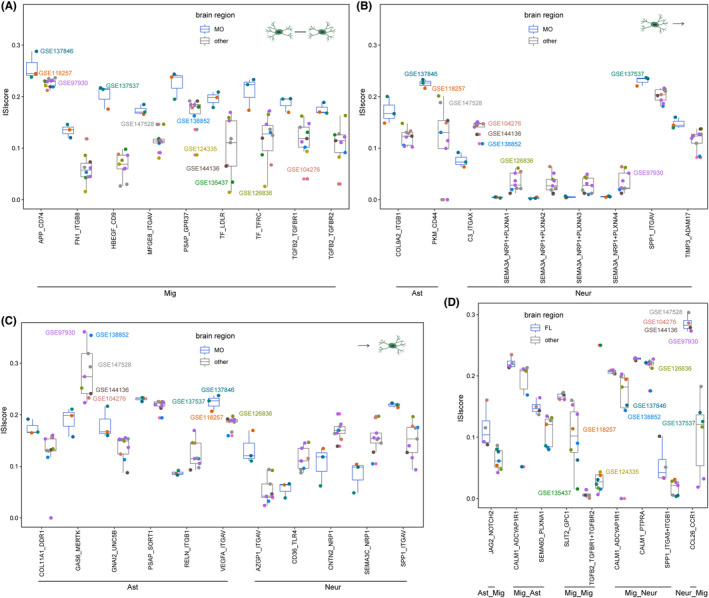
Human region‐specific cell communications. (A) Region‐specific ligand–receptor pairs for (A) microglia as sending and receiving cells. (B) Microglia as sending cells (C) microglia as receiving cells. (D) Significant ligand–receptor pairs associated with microglia between FL and other regions. The colors of the boxes represented the brain regions and the colors of the dots represented the datasets.

Microglia exhibited strong region‐dependent transcriptional properties,^42^ and we further analyzed the region‐specific cell communications in which microglia were involved. For microglia–microglia communications, many ligand–receptor pairs displayed higher score in the meninges and other extracerebral regions (MO) than other brain regions (Figure 5A). In microglia–neuron communications, the ligand SEMA3A and receptor NRP1 + PLXNA1‐4 complex displayed higher score in other regions than MO. In contrast, ligand–receptor TIMP3_ADAM17 displayed higher score in the MO region (Figure 5B). In particular, the ligand–receptor SPP1_ITGAV was regionally distinct not only from microglia to neurons but also from neurons to microglia, where it displayed higher score MO region. And, ligand–receptor pairs CD36_TLR4, CNTN2_NRP1, and SEMA3C _NRP1 displayed lower score in MO for communications from neurons to microglia. Similarly, there existed regional heterogeneity in the communications between astrocytes and microglia, e.g. GAS6_MERTK, RELN_ITGB1, etc (Figure 5C). We also performed the frontal lobe (FL) and other regions and identified region‐specific ligand–receptor pairs for microglia‐related cell communications (Figure 5D).

### 3.6 BrainCelnt platform interface

We provided a user‐friendly resource named BrainCelnt, which allowed users to browse and explore cellular communications in the human or mouse brain under different circumstances. BrainCelnt provided two main modules, ‘Browse’ and ‘Statistics’. The ‘Browse’ allowed users to search ligand–receptor pairs based on species, disease, sex, brain region, and cell type. Corresponding result provided the sub‐dataset ID (BCI ID in BrainCelnt platform), GEO ID, cell type, clinical information, and ligand–receptor scores from four methods. Users can search for ligand–receptor pairs of interest in the search box at the top right, or download the whole table to obtain ligand–receptor scores. In addition, the ‘Statistics’ section allowed users to view clinical statistics of all datasets, including cell type, disease, sex, and brain regions.

## 4 DISCUSSION

In this research, we systematically explored the cell communications within the brain from a single‐cell perspective, by integrating the results of multiple interaction evaluated methods. The integration of different interaction methods can to some extent eliminate their errors and compensate for their shortcomings among them. At the same time, the multiple datasets comparison provided more robust results for cell communications. After comprehensive analysis, we identified many cell–cell interactions involved in normal and disordered brain, and these results provided in‐depth insights into the mechanisms underlying brain immune function and highlight promising genes that may be targeted in the therapy of neurodegenerative diseases.

For calculating the integrated interaction score, we utilized a total of four different methods, which were representative and effective verified by previous studies. For example, CellChat and SingleCellSignalR methods were shown to be more robust to the noise present in the data and ligand–receptor relationships^43^; and CellChat, ICELLNET, and SingleCellSignalR were considered the top three most stable methods.^44^ All these methods were developed to infer cellular interactions based on expression alignment. In addition, we included iTALK in our integration analysis as a way to balance the large differences in methods. It was a tool based on the differential assembly to infer interactions by collating significantly interacting ligand–receptor relationships. Also, it was considered as a stable method for ligand–receptor interactions.^44^ There were also some methods such as CellPhoneDB, which was robust to noise, but we abandoned this method because of the similarity and inferiority of its ligand–receptor database to CellChat. Another method was CellTalker, which differed from iTALK only in downstream analysis and has not been mentioned in previous evaluations, so we gave preference to iTALK. In addition, there were some web‐based algorithms such as CCCExplorer,^45^ which were very demanding in terms of experimental time, and we had to abandon their inclusion in the integration of the method due to a large amount of scRNA‐seq and snRNA‐seq data and condition‐specific sub‐datasets. And, more methods considered in further study could provide the possibility to improve the reliability of ISIcore.

In this study, many condition‐specific ligand–receptor pairs were identified. For example, BMP family‐related interactions were found to be relatively weak in AD pathology, which was reflected by the communication between astrocytes and OPCs. As stated in previous study, defects in the BMP pathway or its regulation were the basis for a variety of human diseases.^46^ Another example, the ligand–receptor SEMA4A_NRP1, which displayed higher score in AD, was mainly manifested related to neurons. In addition to this, disease differences in cell communications were also observed in mouse datasets. Between multiple cell types, there was an enrichment in the strength of interactions between relationship pairs for AD or normal. Obviously, as in the case of FGF1_FGFR2, more potent cell communications were shown in the AD samples (Figure S3). Similarly, considering sex and region factors, we also identified many ligand–receptor pairs that interact differently. For example, SEMA3C_NRP1, which is involved in angiogenesis,^47^ has not been studied related to sex or region.

To explore data heterogeneity issues in the cell communication results, we further took one specific ligand–receptor pair, MLLT4_PVRL3, as an example. Two kinds of comparisons were performed, including two sub‐datasets derived from the same dataset (GSE138852‐AD and GSE138852‐normal), and two sub‐datasets derived from different datasets (GSE138852‐AD and GSE118257). Based on these two sub‐datasets mentioned above, we first performed the combat algorithm for removing the batch effect. Then, we further calculated the integrated ISIscore of this ligand–receptor for all cell communications based on the sub‐datasets after combat analysis. Finally, the correlation analysis of two results from before and after combat was performed. As shown in Figure S6, there existed strong positive correlation between these two results for MLLT4_PVRL3 pair, showing the reliability of previous analysis.

There also exist some limitations in the current study. This study is a hypothesis‐driven study based on scRNA‐seq or snRNA‐seq data. Some key ligand–receptor pairs proposed in this study have already been uncovered in several previous studies. However, there has not yet been biological corroboration of the functions of key ligand–receptor markers identified in this study. In addition, spatial transcriptomic and in situ sequencing have been recently used for exploring the cellular vulnerability and cell communications involved in AD.^48^ Thus, we will specifically add the brain spatial transcriptomic and in situ sequencing data sets to enable more functional interpretation of ligand–receptor interactions. In the meantime, we plan to include additional high‐quality scRNA‐seq and snRNA‐seq datasets and implement visualization capabilities. In current study, the BrainCelnt (http://bio‐bigdata.hrbmu.edu.cn/BrainCelnt) will be a valuable resource for exploring the mechanisms of brain cell communications and neurodegenerative disease prediction.

## 5 CONCLUSION

We systematically explored the cell communications involved in brain by analyzing a total of 28 human and mouse scRNA‐seq and snRNA‐seq datasets. And all these datasets were divided into 71 sub‐datasets when further considering disease, sex and region information. In the meanwhile, we integrated four methods to infer ligand–receptor interaction score and the disease‐specific ligand–receptor was identified for Alzheimer's disease. Furthermore, we explored the sex‐ and region‐specific cell communications, finding that WNT5A‐ROR1 between microglia displayed higher score in males, and SPP1‐ITGAV displayed higher score in the meninges from microglia to neurons. And then, we applied the AD‐specific cell communications for AD prediction model construction and disease subgroup analysis. Finally, a comprehensive platform named BrainCelnt was constructed for researchers to explore the disease‐, sex‐, or region‐specific cell communications in the brain at the single‐cell level.

## AUTHOR CONTRIBUTIONS

Chunlong Zhang, Guiyuan Tan and Xiaoling Zhong analyzed and interpreted the data. Chunlong Zhang, Guiyuan Tan, Yanjun Xu, Yuxi Zhang and Yunyi Peng performed the bioinformatics analyses. Qian Cheng, Xiaoling Zhong, Guiyuan Tan and Yunpeng Zhang performed the biological evaluation. Chunlong Zhang, Yanjun Xu and Feng Li wrote the manuscript. All authors read and approved the final manuscript.

## FUNDING INFORMATION

This work was supported by the National Natural Science Foundation of China (Grant Nos. 62172131, 62101164).

## CONFLICT OF INTEREST STATEMENT

The authors declare that they have no competing interests.

## Supporting information


Figure S1

Figure S2

Figure S3

Figure S4

Figure S5

Figure S6

Table S1

Table S2

Table S3

Table S4

Table S5

Table S6

Table S7

Table S8

## Data Availability

The datasets analyzed in current study are available in Gene Expression Omnibus (https://www.ncbi.nlm.nih.gov/geo/), and we also developed a platform, BrainCelnt (http://bio‐bigdata.hrbmu.edu.cn/BrainCelnt), for users exploring brain condition‐specific cell communications.
