# Supplementary figures and images for: Comprehensive analyses of brain cell communications based on multiple scRNA‐seq and snRNA‐seq datasets for revealing novel mechanism in neurodegenerative diseases
Source: CNS Neurosci Ther. 2023 Jun 2;29(10):2775–86. doi: 10.1111/cns.14280 (PMC10493674; doi:10.1111/cns.14280)

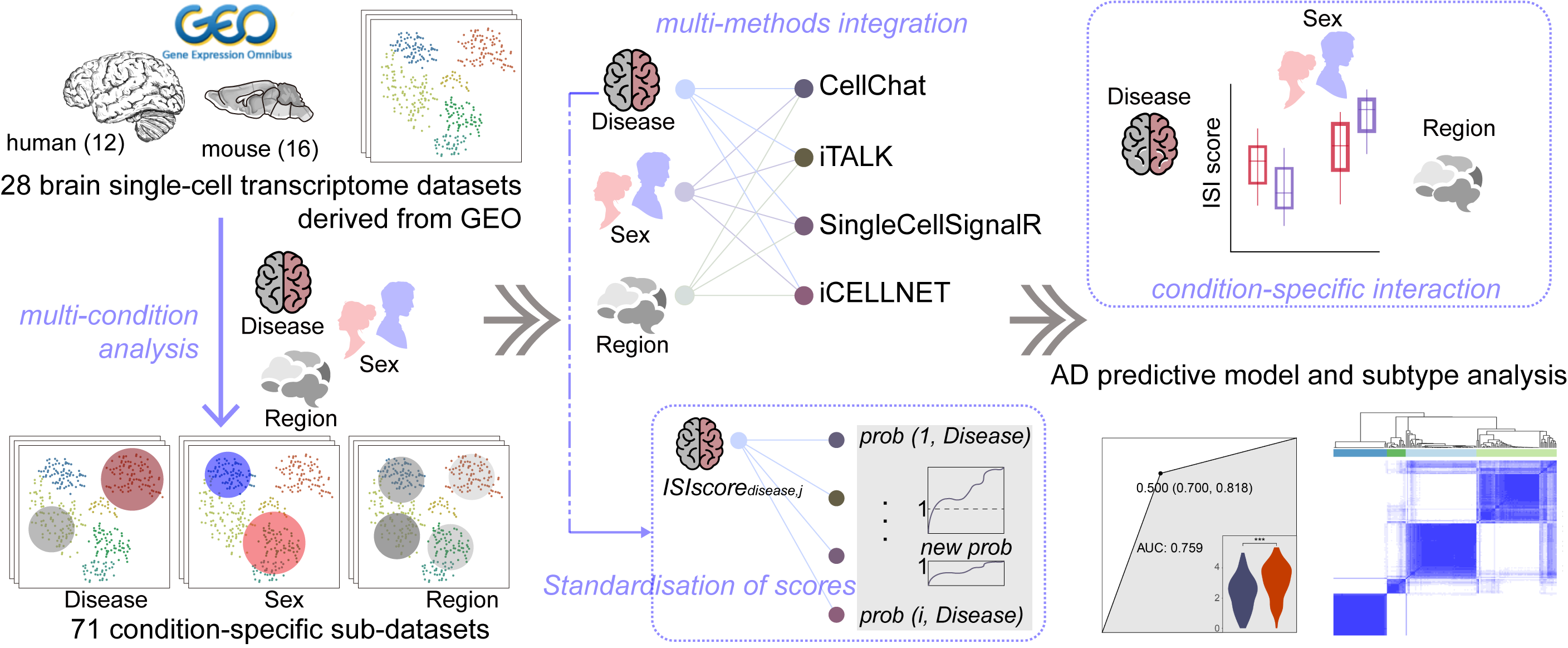

Supplement: Supplementary file 1 — Figure S1 [file CNS-29-2775-s008.tif]

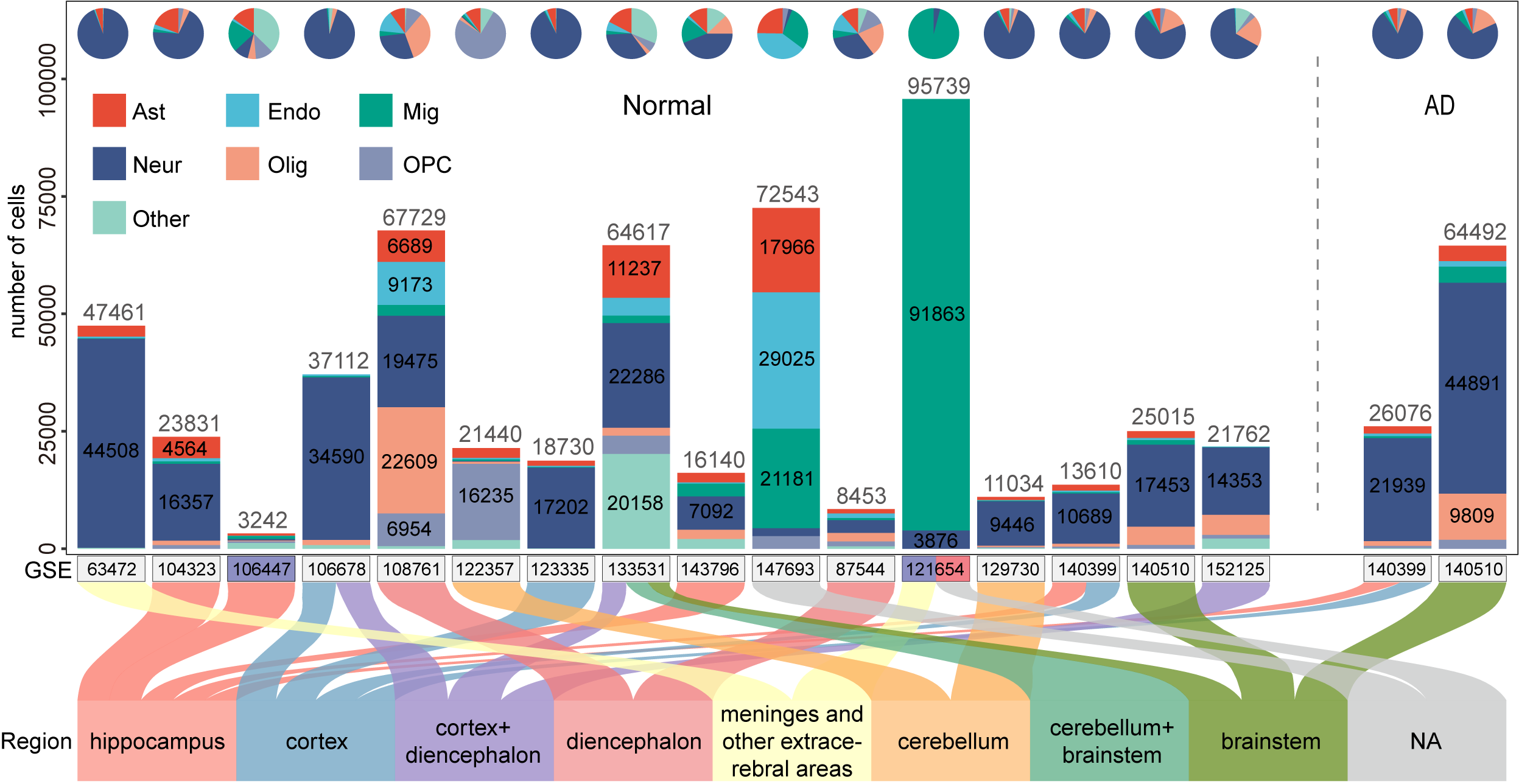

Supplement: Supplementary file 2 — Figure S2 [file CNS-29-2775-s012.tif]

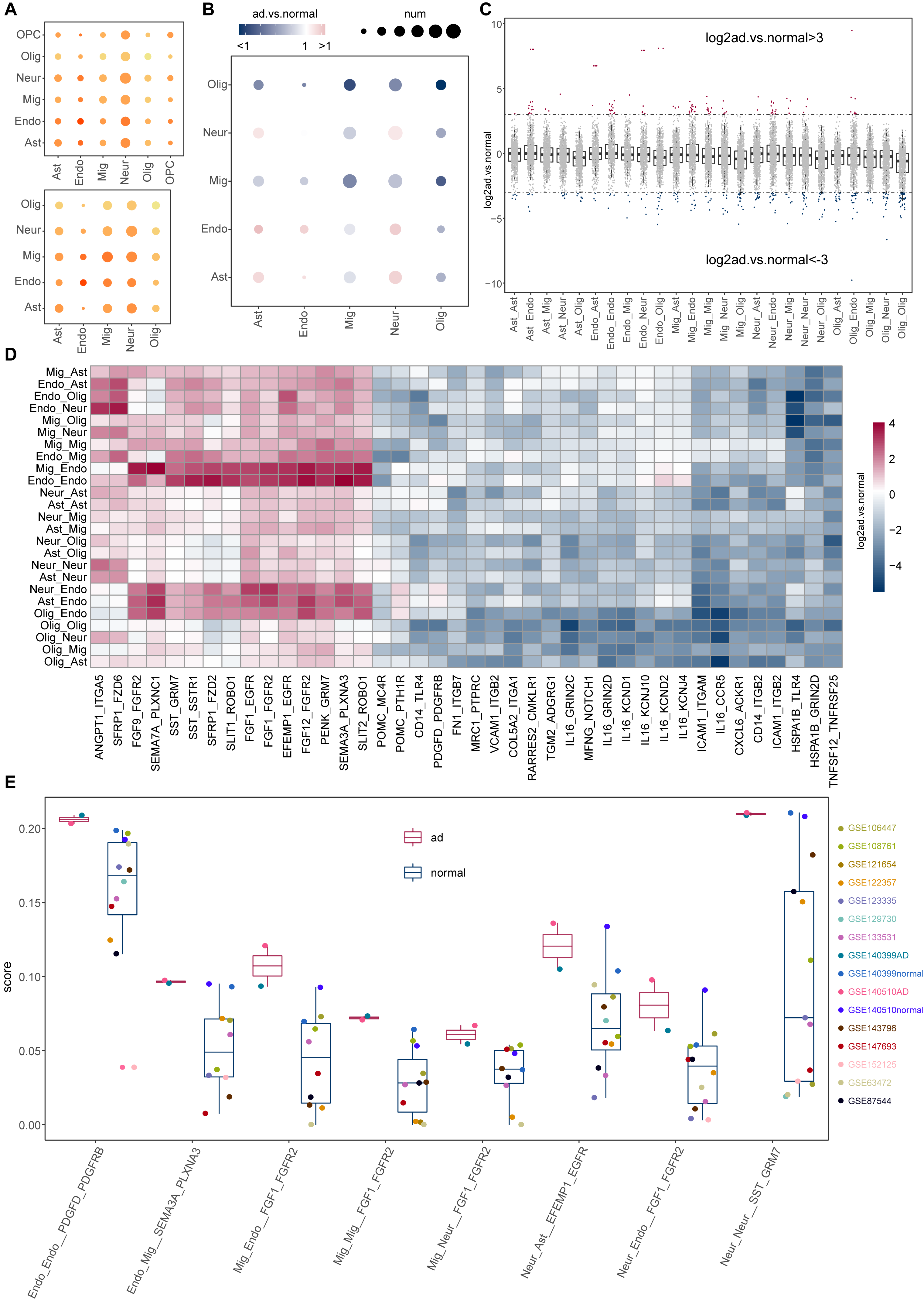

Supplement: Supplementary file 3 — Figure S3 [file CNS-29-2775-s011.tif]

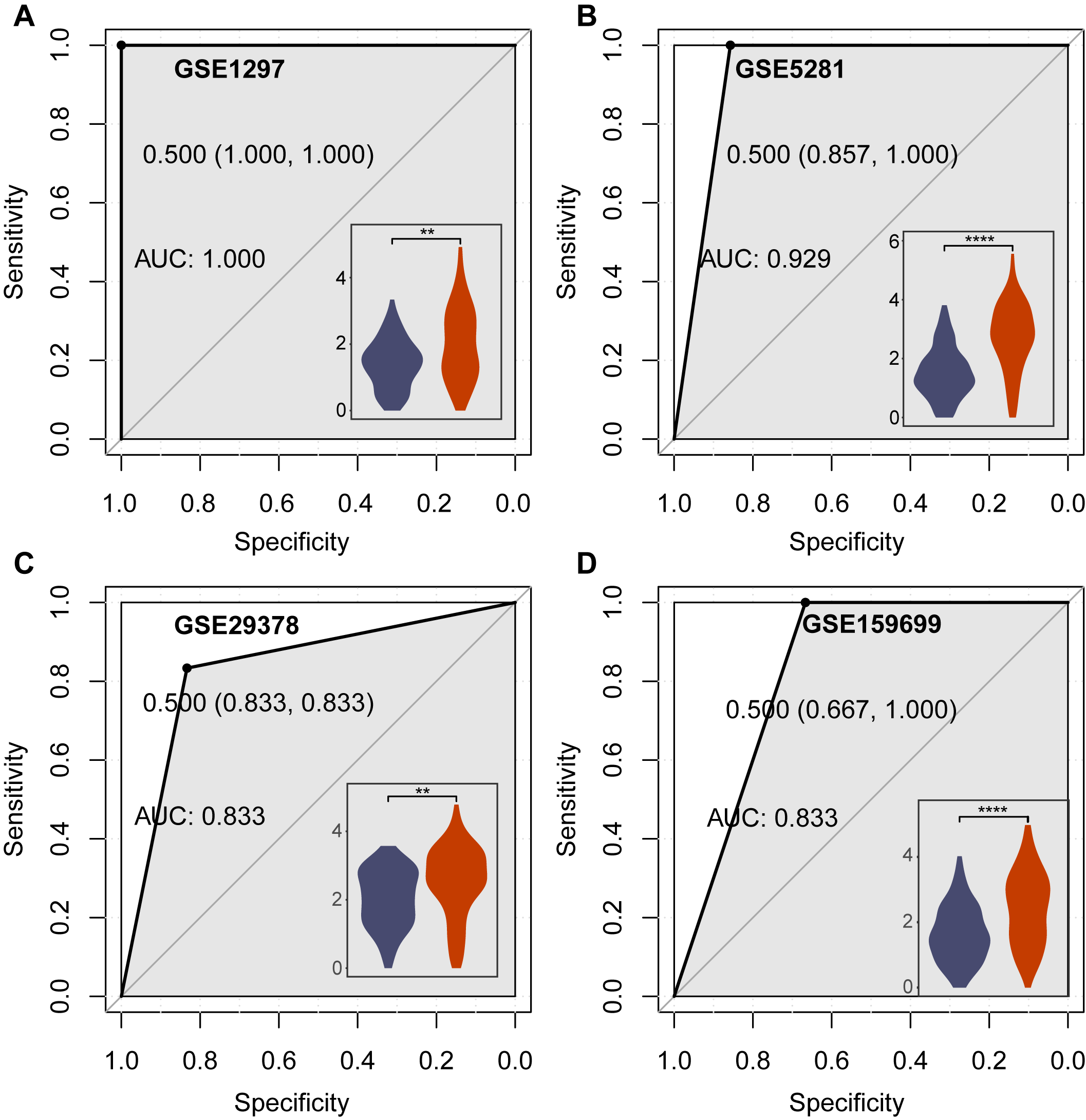

Supplement: Supplementary file 4 — Figure S4 [file CNS-29-2775-s004.tif]

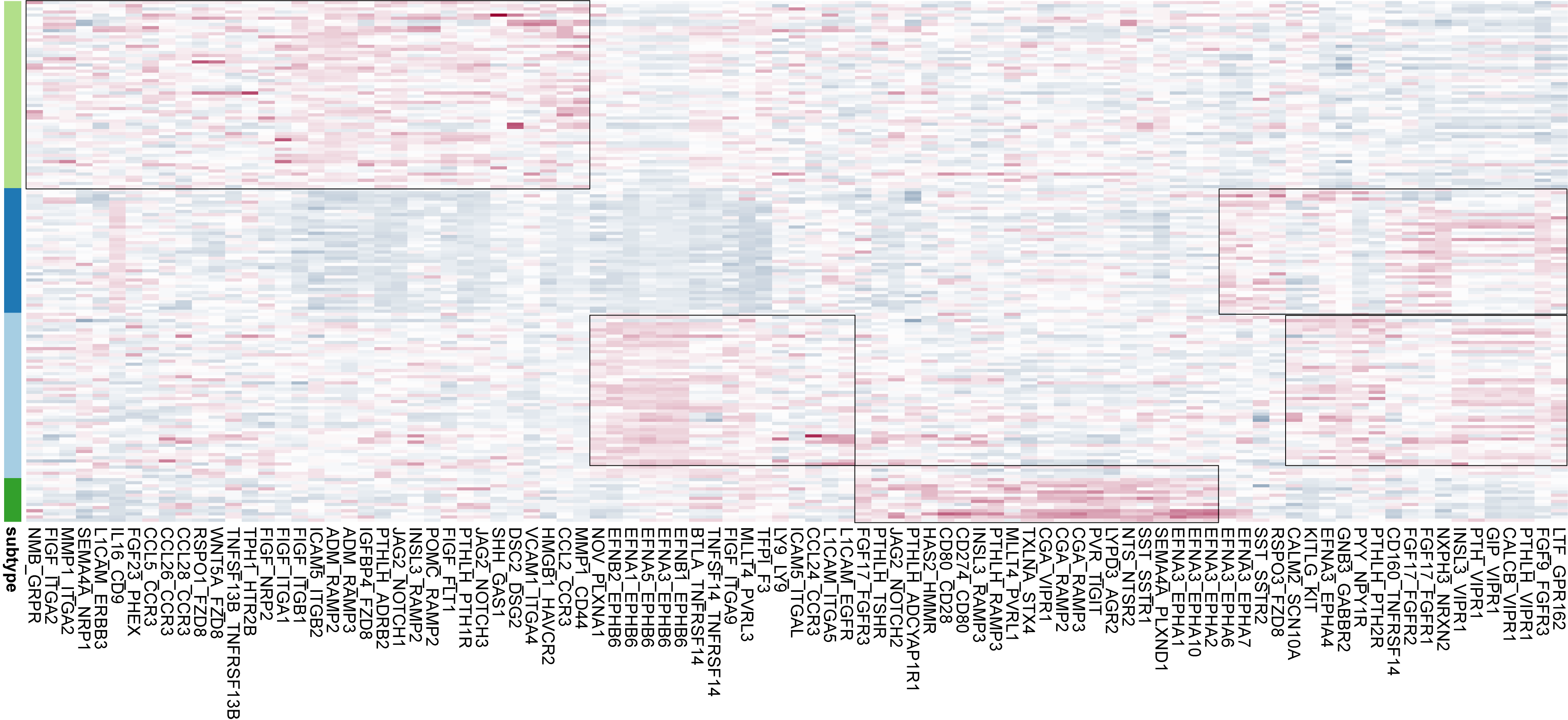

Supplement: Supplementary file 5 — Figure S5 [file CNS-29-2775-s014.tif]

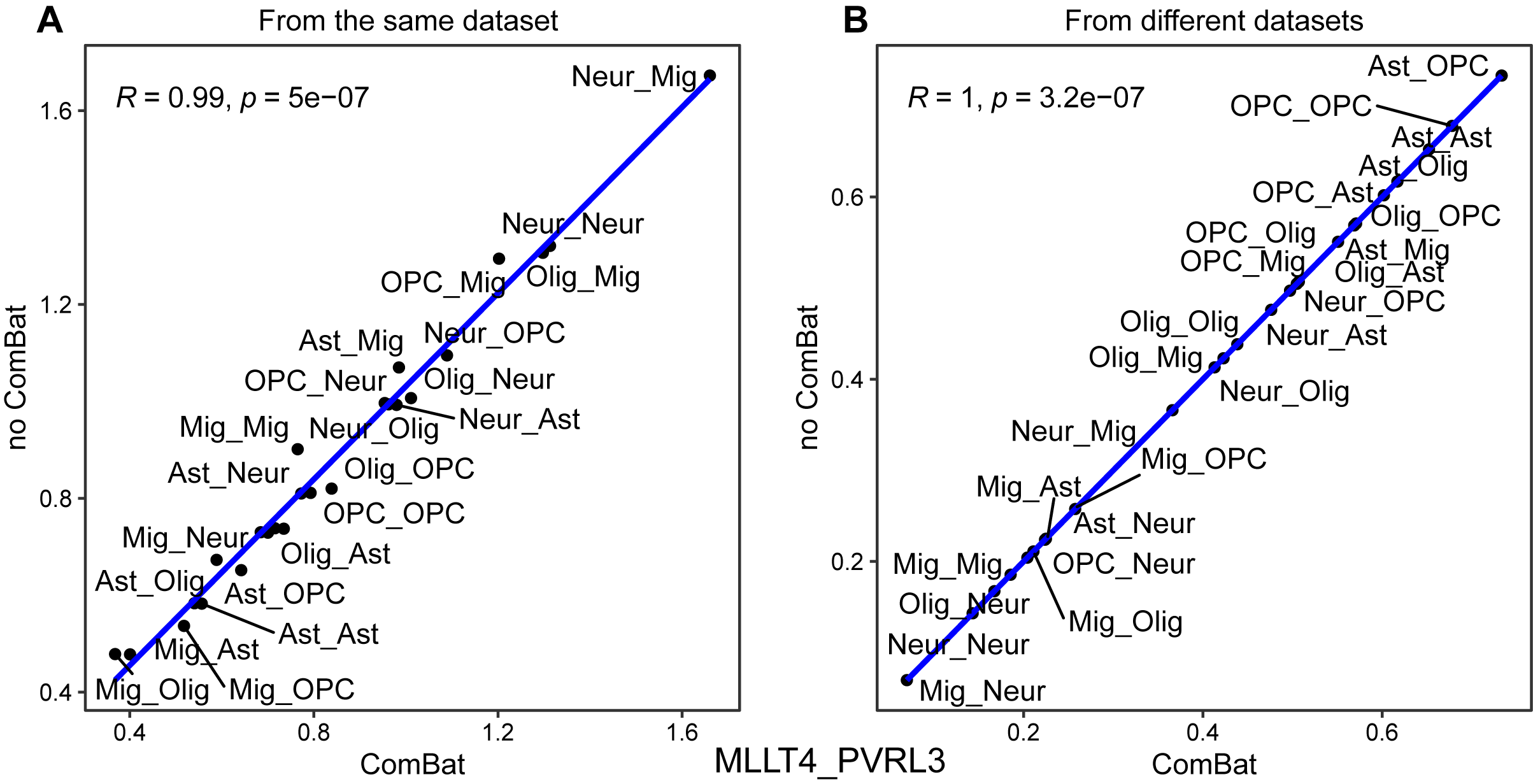

Supplement: Supplementary file 6 — Figure S6 [file CNS-29-2775-s005.tif]
